# Effect of Various Thermal Processing Methods on the Sensory, Textural, and Physicochemical Characteristics of Foal Meat

**DOI:** 10.3390/molecules29225464

**Published:** 2024-11-20

**Authors:** Renata Stanisławczyk, Jagoda Żurek, Mariusz Rudy, Marian Gil, Anna Krajewska, Dariusz Dziki

**Affiliations:** 1 Department of Agricultural Processing and Commodity Science, Institute of Food and Nutrition Technology, College of Natural Sciences, University of Rzeszow, Zelwerowicza 4, 35-601 Rzeszów, Poland; mrudy@ur.edu.pl (M.R.); mgil@ur.edu.pl (M.G.); 2 Department of Finance and Accounting, Institute of Economics and Finance, College of Social Sciences, University of Rzeszow, Cwiklinskiej 2, 35-601 Rzeszów, Poland; jzurek@ur.edu.pl; 3 Department of Thermal Technology and Food Process Engineering, University of Life Sciences in Lublin, Głęboka 31, 20-612 Lublin, Poland; anna.krajewska@up.lublin.pl

**Keywords:** chemical composition, foal meat, physicochemical properties, sensory profile, sous-vide, textural profile

## Abstract

Previous research on the impact of various heat treatment methods on meat has primarily focused on conventional meats from common livestock animals, with limited studies on the effects of different heat treatments on foal meat. The aim of this study was to evaluate how different heat treatments, including sous-vide, affect the sensory, textural, and physicochemical characteristics of foal meat. This allowed for the identification of the most effective heat treatment method to preserve the optimal quality attributes of foal meat while enhancing sensory and textural qualities preferred by consumers. Samples of m. *longissimus thoracis* were subjected to traditional cooking in two variants: in water at 100 °C in a foil bag for 1.5 h, and cooked to a core temperature of 85 °C (16 half-carcasses × 2 traditional heat treatment methods = 32 samples). Additionally, m. *longissimus thoracis* was subjected to sous-vide at 55 °C and 65 °C for 4 and 24 h (16 half-carcasses × 2 temperature settings × 2 time settings = 64 samples). Chemical composition, physicochemical properties, color parameters, hem pigment levels, texture characteristics, and sensory properties were analyzed. Compared to sous-vide samples, traditionally cooked foal meat exhibited higher weight loss (*p* = 0.002), higher fat content (*p* = 0.003), and lower water content (*p* = 0.03). Significant improvements in tenderness were observed in sous-vide samples, with the lowest shear force values (52.86 N/cm^2^ at 55 °C and 48.39 N/cm^2^ at 65 °C; *p* = 0.001) and meat hardness (102.44 N and 101.27 N, respectively; *p* = 0.015) after 24 h of thermal processing. Moreover, sous-vide cooking significantly improved sensory properties of foal meat, particularly juiciness (*p* = 0.002), tenderness (*p* = 0.002), and flavor desirability (*p* = 0.03), which were highly rated by consumers.

## 1. Introduction

In 2022, Poland slaughtered 16,000 horses, yielding a total of 9.3 thousand tons of horse meat. Of these, 12,800 horses were designated for industrial slaughter, producing 7.3 thousand tons of live weight [1]. The production of foal meat in Poland is limited, as these animals are primarily bred for reproduction. Meat from this age group is considered scarce on global markets and commands premium prices. In Poland, foals make up about 10% of the slaughter stock, providing high-quality meat that is largely exported as carcasses or cuts. Countries like Italy, France, and Belgium have a strong preference for foal meat over other types of meat [2]. Foal meat is recognized for its excellent flavor and nutritional qualities. With a low fat content, it serves as a dietary option and contains anti-atherosclerotic substances [3,4]. Importantly, this type of meat has a lower cholesterol level compared to lean cuts of beef or pork, with values of 62 and 71 mg/100 g, respectively [5]. As a result, the regular consumption of horse meat may contribute to lowering total cholesterol and LDL cholesterol levels in healthy individuals [6]. Moreover, this meat demonstrates good water retention, good juiciness, and a bright color [2]. The protein content in meat from foal carcasses is notably high and includes a comprehensive array of essential amino acids, exceeding that of beef in lysine, leucine, tryptophan, phenylalanine, and methionine levels [3,4,7,8].

Among various thermal processing methods for food, sous-vide (SV) cooking is considered the optimal technique for achieving desirable technological and functional attributes in food products [9]. This method enhances high nutritional value, improved tenderness, and juiciness in the finished products [10,11]. Recognized as a contemporary culinary technique, SV establishes new trends in gastronomic technology. The term “sous-vide” is derived from French, meaning “in vacuum” [12,13]. The SV method is a cooking technique performed in a water bath that involves heating high-quality food products (primarily meat) at a precisely regulated temperature for a specified period while they are vacuum-sealed in thermally stable, multi-layer barrier bags made from PA/PE film [13,14,15,16]. After cooking, the products undergo rapid cooling before being stored under refrigeration [17,18,19], which allows for a shelf life of 3 to 5, and even up to 7 weeks prior to reheating and consumption [17]. The pressure created during sous-vide cooking with saturated steam ensures that the meat does not come into direct contact with the heat source, helping to maintain cellular structure, reduce protein–protein interactions and gelation, and improve water retention capabilities [9,20]. This method enhances the sensory qualities and texture of the meat but also extends its shelf life by inhibiting the growth of aerobic bacteria and preventing lipid oxidation [19]. Consequently, the literature consistently identifies sous-vide cooking as one of the most effective thermal processing methods for producing high-quality meat products, particularly when compared to traditional techniques such as grilling, frying, and roasting [21]. In the case of meat, sous-vide thermal processing is based on various combinations of time and temperature. Generally, culinary experts recommend temperature and time settings for sous-vide preparation of beef, pork, or lamb that range from 58 to 63 °C for about 10 to 48 h [22].

Research indicates that sous-vide cooking has been shown to enhance the quality of beef [23], lamb [24], poultry [25], and pork [26]. It is noteworthy that earlier studies focusing on the impact of various thermal processing methods have predominantly examined conventional meats from primary livestock species, such as swine, bovine, and poultry. In contrast, there is limited comprehensive research comparing the effects of different heat treatment methods on the quality of foal meat. Hence, this study aimed to investigate how different heat treatment, including sous-vide, affect the sensory and textural profile, chemical composition, and physical properties of foal meat. This research will help identify the most effective thermal processing method that preserves the optimal quality characteristics of this meat while achieving the sensory and textural properties that consumers find most appealing.

## 2. Results and Discussion

### 2.1. Chemical Composition

Table 1 presents the chemical composition of foal meat based on the applied thermal processing methods. Foal meat, regardless of the sous-vide cooking technique, exhibited a notably lower fat content (*p* = 0.003) and a significantly higher water content (*p* = 0.03) compared to traditional cooking. The choice of thermal treatment had no significant impact on the protein content (*p* = 0.25) in the tested material. The increased water content in meat processed using the sous-vide method can be attributed to the vacuum packaging, which acts as a physical barrier against water loss [27,28]. Conversely, the increased water loss observed in samples subjected to conventional heat treatment leads to a higher concentration of fat. This may be a consequence of the high temperatures causing protein denaturation in muscle tissue during cooking, resulting in a reduction in water retained within the protein structures [29]. This phenomenon refers to chemical composition data of horse meat discussed in this study.

The study by Sujiwo et al. [30] on horse meat found no significant differences in water and fat content between the control group (meat cooked at 72 °C for 40 min) and samples subjected to sous-vide cooking (65 °C and 70 °C for 12, 18, and 24 h). However, sous-vide samples generally had a significantly higher protein content compared to the control group (26.38%), except for those cooked at 65 °C for 12 h (28.14%). The protein content was shown to be influenced by the cooking temperature. A study by Głuchowski et al. [31] on chicken breast (musculus pectoralis major) found that thermal processing methods decreased water content while increasing protein and fat levels. Samples cooked in a pot to a core temperature of 70 °C, steamed to 75 °C, and those processed under higher sous-vide conditions at temperature 66 °C and 75 had lower water content compared to those treated at the lowest evaluated temperature (SV64).

The aforementioned research results [30,31] indicate an increase in protein content in heat-treated meat. However, the results presented in this study do not show a significant effect of the applied heat treatment on the protein content in the tested meat.

### 2.2. Physicochemical Properties

The evaluation of substances that react with 2-thiobarbituric acid to produce a color change is a common technique for assessing lipid oxidation levels in meat. The findings are expressed as malondialdehyde content, used as an indicator of TBARS. This study indicated that the method of thermal processing had a significant impact on the malondialdehyde levels (*p* = 0.03) in the studied samples (Table 2). Foal meat samples cooked at 100 °C for 1.5 h exhibited the highest levels of malondialdehyde, surpassing 1 MDA/kg in comparison to all other meat samples analyzed. When traditional thermal processing was applied to achieve an internal temperature of 85 °C in the meat samples, the TBARS values obtained were similar to those of samples processed using sous-vide methods, regardless of the specific temperature or duration of treatment. Increasing the duration of sous-vide thermal processing from 4 to 24 h led to an elevation in the value of the analyzed indicator across both temperature variants; however, this trend was not statistically validated (*p* = 0.1). It is important to emphasize that the distribution of results presented in this study is significantly affected by the type of raw material under investigation. Foal meat is characterized by a high content of polyunsaturated fatty acids (PUFAs) [32,33], which are particularly prone to oxidation [34,35]. Mild lipid oxidation during the initial phases of cooking meat can enhance desirable aromas; however, lipid autooxidation may lead to off-flavors and rancidity, frequently described as “warmed-over flavor” [36]. As a result, oxidation reactions play a vital role during thermal processing because they influence the characteristics of this type of meat. A previous study [37] indicated that the value of the analyzed indicator increased with higher temperatures and longer durations of sous-vide cooking. Haghighi et al. [38] found that raising the sous-vide cooking temperature from 60 °C to 100 °C for chicken breast fillets led to a significant increase in TBARS values, which rose from 0.29 mg MDA kg^−1^ to 2.91 mg MDA kg^−1^. Additionally, the same authors reported that prolonging the sous-vide cooking time from 60 min to 150 min resulted in an increase in TBARS values from 0.29 mg MDA kg^−1^ to 0.94 mg MDA kg^−1^, even when maintaining a constant temperature of 60 °C. Sánchez del Pulgar et al. [28] found greater TBARS values in cooked pork cheeks samples at 60 °C for 12 h; however, lower values were observed in samples subjected to 80 °C for the same duration.

The results presented in Table 2 indicate that the type of thermal treatment applied does not significantly affect the oxidation–reduction potential (ORP) or water activity in foal meat. The ORP values ranged from 339.50 to 392.20 mV, while water activity levels were between 0.990 and 0.998. Stanisławczyk et al. [37] reported ORP values ranging from 342.30 to 392.40 mV for sous-vide-processed horse meat, showing that these values are independent of the cooking temperature and time. Similarly, the water activity of sous-vide-cooked horse meat samples ranged from 0.978 to 0.995, with values for this parameter also remaining unaffected by the cooking conditions.

Cooking loss is determined by the mass transfer occurring during thermal processing, indicating that different cooking techniques will yield varying degrees of loss [39]. Table 2 presents the percentage weight loss of foal meat samples subjected to various thermal processing methods, including both water-based cooking and sous-vide technology. The highest weight losses, ranging from 39.04% to 41.19%, were observed in meat cooked using traditional methods in water pots. In this study, the weight loss of meat prepared using sous-vide technology was statistically significantly lower than that observed for traditionally cooked meat (*p* = 0.002). The least weight loss, approximately 13%, was noted in samples subjected to lower temperatures (55 °C) for 4 h. As the temperature and cooking time increased during sous-vide preparation, the percentage of weight loss also increased. These findings can be attributed to elevated temperatures that induced the denaturation of myofibrillar proteins and the actomyosin complex, leading to muscle contraction. This denaturation process not only causes structural alterations but also promotes the release of fluids from muscle fibers, diminishing water-holding capacity and consequently increasing cooking losses. The sous-vide process, involving vacuum-sealed foal meat, resulted in reduced weight loss. The increased weight loss observed during conventional cooking is attributed to evaporation. Vacuum packaging effectively inhibited water evaporation from the meat samples, acting as a physical barrier that retained moisture within the packaging while also limiting liquid discharge due to constrained space. Increasing the temperature and duration during sous-vide cooking leads to greater weight losses due to the pressure differential between the meat product and its surroundings [40]. The obtained research findings are consistent with the results of other studies regarding the impact of thermal treatment on weight loss in equine meat [30,37], poultry [11,38,41], beef [24,27], and pork [28].

### 2.3. Color Parameters and the Level of Heme Pigments

Table 3 presents the color parameters and heme pigment levels in foal meat as affected by the type of thermal processing applied. The analysis of lightness (L*) reveals that the thermal treatment method significantly influenced this parameter (*p* = 0.02). Traditional cooking methods led to a significant decrease in lightness of the meat samples, while sous-vide cooking notably increased lightness compared to traditional methods. The highest L* values for foal meat were observed in samples cooked sous-vide for 4 h, across all temperature ranges tested in this study. There were no statistically significant differences between any of the sous-vide treatments.

The higher L values observed in meat cooked sous-vide at lower temperatures, compared to those cooked using conventional methods, may be attributed to the increased amount of free water on the surface of the sliced sample prior to color measurement [42]. Sous-vide samples cooked at lower temperatures retained more moisture, which resulted in water being released onto the surface during slicing for color analysis. In contrast, samples that experienced greater moisture loss during conventional cooking showed lower water content and visible exudate on the surface [24]. Sujiwo et al. [30] observed significantly higher L values in horse loin samples treated at 65 °C for 24 h compared to those cooked at 70 °C for 18 h. The distribution of results was notably influenced by both the cooking temperature and duration.

The type of thermal treatment applied significantly influenced the variation of the a* color parameter in foal meat. The highest levels of red color (a*) were observed in samples subjected to sous-vide cooking at 55 °C. Increasing the sous-vide cooking temperature to 65 °C resulted in a significant decrease in this parameter (*p* = 0.003) due to more intense pigment denaturation, indicating that myoglobin degradation increases with cooking temperature. Denaturation of myoglobin leads to a reduction in the red coloration of meat. This process typically occurs at approximately 60 °C. Furthermore, prolonging the sous-vide cooking time resulted in a slight decrease in the average a* color values across all tested temperature ranges, though these differences were not statistically significant. The lowest a* color parameter values in foal meat were observed in samples subjected to traditional cooking at 100 °C in a foil bag for 1.5 h, as well as in those cooked until the geometric center reached 85 °C. Other research has indicated a reduction in redness corresponding to higher cooking temperatures for beef [43], pork [28], and lamb [24]. The degree of myoglobin denaturation has been shown to correlate with cooking temperature, resulting in a greater extent of myoglobin denaturation and subsequently leading to lower a* values [44,45]. In the study by Sujiwo et al. [30], both cooking temperature and cooking time significantly influenced the distribution of a* values in the tested groups of horse meat samples. The recorded a* values in the group subjected to 70 °C for 18 h (13.84) and 70 °C for 24 h (13.60) were considerably lower than those in the control groups exposed to 75 °C (16.95) and 65 °C for 12 h (16.29) [30]. It is important to note that the results obtained for the color parameter a* in this study (Table 3) differ from those reported by Christensen et al. [46]. The authors found that as the temperature in the LTLT cooking method increased from 53 °C to 58 °C, the intensity of the brown color increased, while the pink color diminished in *Semitendinosus* (ST) from young bulls (12–14 months), *Semitendinosus* (ST) from slaughter pigs, and *Pectoralis profundus* (PP) from chickens.

The yellow color component (b*) in foal meat samples indicates that the type of thermal processing employed does not have a significant impact on the values of this parameter. Across all cooking methods, the yellow color component (b*) for the samples varied between 12.51 and 13.88. However, the existing literature emphasizes a discernible trend toward a reduction in a* values coupled with an increase in b* values during prolonged sous-vide cooking durations [24,43,47]. This observed phenomenon can be attributed to the formation of metmyoglobin and its subsequent heat-induced denaturation, which ultimately results in a brownish hue. In contrast, Sujiwo et al. [30] demonstrated that the distribution of b* values in loin horse samples subjected to sous-vide cooking was significantly influenced by cooking duration. Specifically, samples cooked at 65 °C for 24 h (13.91) and at 70 °C for 24 h (14.31) exhibited markedly elevated b* values compared to the control group, which was cooked at 75 °C for 40 min.

The primary heme pigment responsible for meat color is myoglobin. Moreover, color changes in meat during thermal processing are influenced by other pigments, including deoxymyoglobin, oxymyoglobin, sulfmyoglobin, and metmyoglobin. Myoglobin forms undergo degradation during thermal processing through oxygenation, oxidation, and reduction reactions, which consequently influence the color of the meat [48]. The conducted research indicates that the type of thermal treatment significantly affects the levels of myoglobin (Mb) (*p* = 0.01) and the overall content of heme pigments (OZB) in foal meat. Samples cooked traditionally at 100 °C in a foil bag for 1.5 h and those cooked until reaching 85 °C at the geometric center exhibited significantly higher levels of Mb (*p* = 0.01) and OZB (*p* = 0.0001) compared to all other samples analyzed. A notably high concentration of myoglobin was recorded in samples cooked sous-vide at 65 °C for 24 h. In contrast, the lowest concentrations of myoglobin were found in samples cooked sous-vide at 55 °C for both 4 and 24 h. Regarding the overall heme pigment content, the lowest values were observed after 4 h of sous-vide cooking at 55 °C. The type of thermal treatment employed did not significantly affect the levels of metmyoglobin (MMb) (*p* = 0.35) and oxymyoglobin (Mb•O_2_) (*p* = 0.27) in the examined samples. Slightly elevated levels of these pigments were detected in the sous-vide cooking at 55 °C after both 4 and 24 h. An increase in temperature from 55 °C to 65 °C and an extension of sous-vide cooking duration from 4 to 24 h resulted in a slight reduction in the levels of the analyzed pigments, although this relationship was not statistically significant.

The results obtained coincide with those reported in earlier research [37], where it was demonstrated that the temperature used, the duration of sous-vide thermal treatment, and the interaction between time and temperature significantly influenced the levels of pigments in horse meat samples. Stanisławczyk et al. [37] indicated that horse meat cooked sous-vide at 55 °C for 4 h exhibited a high proportion of red color and oxymyoglobin.

### 2.4. Texture Parameters

Table 4 presents the texture parameters of foal meat in relation to the type of thermal processing employed. The results show that the heat treatment method significantly affected the variation in shear force within the examined samples. The shear force values required to cut the longissimus thoracis muscle were notably highest for those subjected to traditional cooking methods in water baths (77.82–74.55 N/cm^2^). In contrast, sous-vide cooking produced significantly lower force values (61.05–48.39 N/cm^2^). Extending the duration of sous-vide treatment from 4 to 24 h across all temperature ranges resulted in a significant reduction in the force required to cut the samples (*p* = 0.001).

Instrumental evaluation of texture parameters indicated that the thermal processing method had a significant impact on the textural attributes of this kind of meat. The highest values for hardness 1 (*p* = 0.015), hardness 2 (*p* = 0.023), stiffness 5 (*p* = 0.0001), stiffness 8 (*p* = 0.034), and chewiness (*p* = 0.0002) were recorded in samples subjected to traditional cooking using two different methods. In contrast, samples cooked sous-vide at 65 °C for 24 h displayed the lowest hardness values. Furthermore, extending the sous-vide cooking duration from 4 to 24 h led to a significant reduction in the measured parameters.

Stiffness, an important parameter of meat texture, quantifies the force needed to deform a sample over a specified distance during compression. The samples analyzed demonstrated significantly greater stiffness (stiffness 5 (*p* = 0.0001), stiffness 8 (*p* = 0.034)) when subjected to traditional cooking methods, while those cooked sous-vide exhibited reduced values. Additionally, prolonging the duration of sous-vide cooking resulted in a substantial decrease in the stiffness of foal meat.

The samples prepared using traditional cooking methods in two variations exhibited significantly greater chewiness (*p* = 0.0002) compared to those subjected to sous-vide cooking. An increase in temperature from 55 °C to 65 °C resulted in a marginal rise in the chewiness of the meat, whereas prolonging the sous-vide cooking duration from 4 to 24 h led to a slight reduction in the values of the analyzed texture parameter, although this trend was not statistically significant.

Sujiwo et al. [30] performed instrumental texture analysis on horse meat and demonstrated that sous-vide cooking reduced the shear force required to slice horse loin. However, no significant differences were observed in hardness and chewiness between the control samples (cooked at 72 °C for 40 min) and those prepared using sous-vide cooking at 65 °C and 70 °C for 12, 18, and 24 h. Notably, the cohesiveness and springiness of samples cooked at 70 °C for 18 and 24 h showed significant differences compared to the control. Springiness was found to be significantly influenced by both cooking time and the interaction between time and temperature during sous-vide processing [30]. Stanisławczyk et al. [49] reported that prolonged sous-vide thermal processing at elevated temperatures improved texture parameters such as shear force and hardness in horse meat. These findings align with those of Roldán et al. [24], who observed that sous-vide cooking enhances the tenderness of lamb loin when cooking times are extended.

The results of this study regarding the texture of foal meat can be attributed to the dissolution of connective tissue caused by the thermal processing temperature, which enhances meat tenderness. However, the denaturation of myofibrillar proteins may also contribute to increased toughness. Meat toughening is further influenced by water loss from muscle tissue during heating. Additionally, texture changes during heating are affected by the transition of muscle tissue from a viscoelastic to an elastic state. The reduction in hardness and the shear force required to slice the meat samples with extended sous-vide cooking times can be attributed to the increased solubility of collagen over longer cooking durations. Myofibrillar shrinkage may have already reached its peak, showing no further significant increase with prolonged cooking times. The extensive breakdown of the perimysium surrounding muscle bundles during prolonged cooking may also explain the increased tenderness observed in foal meat [13]. The temperature applied undoubtedly influenced the distribution of texture-related results. Exposure to temperatures up to 65 °C enhances tenderness by promoting the aggregation of sarcoplasmic proteins into a gel, which improves chewability. Higher temperatures between 65 and 85 °C can result in tougher meat due to an increased elastic modulus [50]. The tenderness of meat cooked at 60 °C for extended periods may be linked to the residual collagenolytic activity observed after 6 h [13].

### 2.5. Sensory Properties

**Figure 1:**
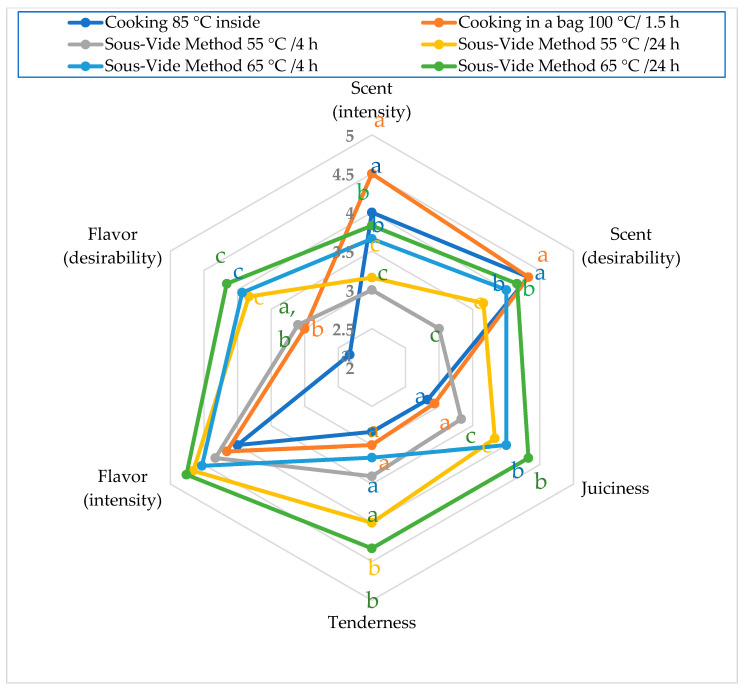
Sensory characteristics of foal meat (points) based on the method of thermal processing; a, b, c—indicate statistically significant differences in values between the types of thermal treatment—*p* < 0.05.

Compared to traditional cooking methods, sous-vide significantly enhances meat texture and sensory qualities by minimizing protein aggregation and gelation, which can contribute to reduced toughness [22,51]. The average values of sensory quality attributes for foal meat, based on the type of thermal treatment, are illustrated in Figure 1. The results of the organoleptic assessment indicate that the type of heat treatment significantly influences the sensory quality. The aroma (intensity and desirability) of samples cooked at 100 °C in a foil bag for 1.5 h and those cooked to a core temperature of 85 °C received the highest scores, ranging from 4 to 4.5 points. In terms of aroma (intensity and desirability), it can be concluded that sous-vide cooking significantly contributes to the reduction in the analyzed sensory quality attributes of meat compared to traditional cooking methods (*p* = 0.003 and, 0.003). Extending the sous-vide cooking time from 4 to 24 h at both 55 °C and 65 °C had no effect on aroma (intensity and desirability) for foal meat. An opposite trend was observed for the other sensory quality attributes. When comparing sous-vide cooking to traditional methods, there was a significant increase in juiciness (*p* = 0.004), tenderness (*p* = 0.004), and flavor (desirability) (*p* = 0.020) of meat. The traditional cooking method, which aimed to reach a core temperature of 85 °C, yielded the lowest scores for the sensory quality attributes assessed. In the case of sous-vide cooking, both raising the temperature from 55 °C to 65 °C and extending the cooking duration from 4 to 24 h improved the juiciness, tenderness, and flavor of the samples.

In terms of taste, flavor, tenderness, and juiciness of chicken meat, Kerdpiboon et al. [51] reported that the most favorable values for these attributes were achieved in meat processed using the sous-vide method at temperatures ranging from 55 to 60 °C for periods of 3 to 5 h. Enhanced juiciness of pork loin processed via the sous-vide method was observed when subjected to temperatures between 60 and 65 °C for a duration of 3 h, which can be attributed to increased moisture content and minimized cooking losses [52]. Polak and Markowska [11] demonstrated that turkey breast meat prepared using sous-vide method at a temperature of 64 °C for 120 min exhibited the most favorable organoleptic qualities, whereas the lowest ratings were assigned to meat subjected to the same temperature for only 60 min. Furthermore, Karpińska-Tymoszczyk et al. [41] indicated that the most desirable flavor and aroma of chicken breast fillets can be achieved by employing the sous-vide method at a temperature of 55 °C for 260 min. The cited authors explain that at low temperatures, such as 55 °C, flavor and aroma can be enhanced through interactions between sugars and amino acids. This phenomenon also leads to the release of flavor compounds in meat due to the thermal degradation of thiamine and lipid oxidation. The flavor and aroma of meat can significantly differ based on the type of meat cut, even among different cuts from the same species, as well as across various meat types and products derived from distinct species. Thathsarani et al. [40] indicate that maintaining the cooking temperature of meat below 70 °C yields a more favorable flavor profile than cooking at higher temperatures. However, this outcome may also be influenced, as previously noted, by the type of meat and the specific cuts involved. Sous-vide cooking effectively minimizes the loss of flavor and aroma associated with volatile compounds in meat, primarily due to the vacuum packaging, which retains these compounds within the sealed environment. In terms of juiciness, extensive research [14,15,41,53] demonstrates that sous-vide cooking at lower temperatures significantly enhances this quality. This improvement can be explained by the fact that, during sous-vide processing at reduced temperatures, denaturation of proteins, contraction of muscle fibers, and dissolution of collagen in the muscle tissue are minimized, resulting in greater moisture retention within the muscle, ultimately leading to increased juiciness of the meat.

## 3. Materials and Methods

### 3.1. Experimental Design

The study material consisted of *longissimus thoracis* muscle samples taken from 16 half-carcasses of foals aged 6 to 12 months, obtained from individual farmers in southeastern Poland. The pre-slaughter weights of the animals ranged between 250 and 320 kg (average 285 ± 20). The foals belonged to the Malopolski and Silesian breeds, which are the most prevalent horse breeds in this region. The foals were randomly selected, with an equal distribution of 50% fillies and 50% uncastrated colts. All the animals were healthy and raised in an extensive farming system. During transport, foals were housed individually in designated pens within livestock facilities for approximately 24 h, under the supervision of veterinary professionals to ensure their welfare. Slaughtering procedures adhered to standard practices in the meat industry and complied with European regulations [54]. Before slaughter, the foals were stunned using a captive bolt pistol. Muscle samples for quality analysis were collected from the longissimus thoracis muscle, located between the 13th and 14th thoracic vertebrae. These samples were subsequently chilled at 4 °C ± 0.5 °C for a period of 10 days to assess the effects of heat treatment on foal meat quality.

### 3.2. Cooking Procedure

Upon arrival at the laboratory, the muscle samples were maintained at a controlled temperature of 3 ± 0.5 °C and evenly divided into six batches, each weighing approximately 200 ± 30 g. A total of 6 foal meat samples were taken from 16 half-carcasses of foals, one for each type of heat treatment, which constituted a total of 96 samples. Four of these batches were placed in mesh foil bags designed for sous-vide heat treatment and vacuum-sealed using a vacuum packer (Inauen, Schwanden, Switzerland). Following the vacuum-sealing process, the meat samples underwent thermal treatment in a water bath (Hendi, Gądki, Poland). They were cooked at 55 °C and 65 °C for either 4 or 24 h, producing a total of 64 meat samples (16 half-carcasses × 2 temperature settings × 2 time settings = 64 meat samples). Following thermal processing, the meat samples were rapidly chilled to a temperature of 4 °C ± 0.5 °C. The remaining two batches, which constituted the control group (32 samples), were subjected to conventional cooking methods. One batch was boiled until the internal temperature at the geometric center reached 85 °C, while the other batch was boiled in vacuum-sealed foil bags at 100 °C for 1.5 h (16 half-carcasses × 2 conventional heat treatment methods = 32 samples). After the heat treatment, all samples were immediately cooled to 4 °C ± 0.5 °C.

### 3.3. Analytical Methods

The moisture content of the samples was measured according to the PN-ISO 1442:2000 standard [55], while protein content was determined using the Kjeldahl method, as outlined in the PN-75/A-04018 standard [56]. Fat content was evaluated using the Soxhlet extraction method in accordance with the PN-ISO 1444:2000 guidelines [57]. Lipid oxidation was measured through the TBARS (2-thiobarbituric acid-reactive substances) index, applying the methodology outlined by Pikul et al. [58]. Oxidation–reduction potential (ORP) was evaluated using an ERPt-13 Hydromet No. 235-type combination electrode connected to a CPC-501 digital pH/conductivity meter (Elmetron, Zabrze, Poland). Once the ORP stabilized, the readings were converted to redox potential values (EH mV) by adding the reference electrode potential (Em = 211 mV at 20 °C) to the measured Em values. Each 10 g sample, previously ground using a laboratory mill with a 3 mm mesh, was homogenized in 50 cm^3^ of distilled water at 20 °C. The process was conducted using an ULTRA TURAX T25 homogenizer (IKA, Staufen, Germany) operating at a spindle speed of 15,000 rpm. The potential of the resulting suspension was measured at 20 °C, with readings taken to an accuracy of 0.01 [59].Water activity was determined using a Novasina AG LabMaster—aw neo meter (Lachen, Switzerland) following the method described by Duma-Kocan et al. [60]. The loss was determined using the formula below [20].

$$Weight\;loss\;(\%)=\frac{weight\;of\;raw\;meat-weight\;of\;cooked\;meat}{weight\;of\;raw\;meat}\;•\;100$$

The color of the meat samples was evaluated using the CIE LAB color system (L*, a*, and b* values) with an NR20XE (3nh Technology Co., Ltd., Shenzen, China). The blooming process was performed in darkness at 4 °C, and color measurements were taken at three locations on each sample after sous-vide cooking and 60 min of cooling at 4 °C, following the methodology outlined by [37]. The proportion of heme pigments in the meat samples was measured according to the method described by Krzywicki [61].

Using a TA.XT plus texturometer (TA-XT plus; Stable Micro Systems Ltd., Surrey, UK) equipped with a triangular-cut Warner–Bratzler shear blade, the shear force was measured. Raw meat samples were taken using a cylinder-shaped cork borer (12.7 mm in diameter) cut along the muscle fibers. The prepared samples were then sectioned, and the shear force (N/cm^2^) exerted during the cutting process was recorded. Each sample underwent three technical repetitions.

Raw meat samples were diced into 20 mm cubes and analyzed for texture using a CT3-25 texture analyzer (Brookfield, WI, USA) with a cylindrical probe, following the method described by Stanisławczyk et al. [62]. The Texture Pro CT software (V.1.9 Build 39; Brookfield, WI, USA) was used to measure various parameters, including hardness, springiness, adhesiveness, cohesiveness, stiffness, resilience, and chewiness. A panel of 15 trained individuals performed the sensory evaluation following ISO 8586:2023 [63] and ISO 8587:2006 [64] standards. Using a 5-point scale, they assessed aroma intensity, taste intensity, aroma desirability, taste desirability, juiciness, and tenderness in a dedicated laboratory, as outlined by Stanisławczyk et al. [49].

### 3.4. Statistical Analysis

All observations composing the experiment (6 types of heat treatment × 16 batches) were included in the statistical analysis. The data were verified for normality using the Kolmogorov–Smirnov test. The homogeneity of variance was verified using the Brown–Forsythe test. Chemical composition, selected physicochemical properties, color parameters, texture, and sensory evaluations of the meat were analyzed by a one-way analysis of variance (ANOVA), which considers type of heat treatment as a fixed effect and batch as a random term. The Statistica 13.3PL software (STATISTICA v. 10; StatSoft, Krakow, Poland) package from TIBCO Software Inc. (Palo Alto, CA, USA) was utilized for this analysis. Tukey’s HSD test was used to evaluate the significance of differences at a significance level of *p* < 0.05. Table 1, Table 2, Table 3 and Table 4 present mean values and standard error of individual qualitative features of meat.

## 4. Conclusions

The sous-vide method emerged as the most effective thermal processing technique for preserving the optimal quality attributes of foal meat, particularly in terms of sensory and textural characteristics preferred by consumers. These favorable qualities were most prominent after 24 h of sous-vide heat treatment. Foal and horse meat exhibit distinct differences from the meat of other animal species, especially in terms of processing and functional properties. This study demonstrates that the best sensory and textural qualities, such as tenderness and juiciness, were achieved in foal meat only after 24 h of sous-vide cooking. In contrast, meat from other species reached the most favorable sensory characteristics with shorter sous-vide processing times, indicating that foal meat requires different culinary preparation. Further studies on the quality of foal meat subjected to sous-vide processing at temperatures above 70 °C would be of interest.

## Figures and Tables

**Table 1:** Chemical composition of foal meat as influenced by different thermal processing methods (means ± SE).

Specification	Type of Thermal Processing						*p*-Value
	Cooking * 85 °C	Cooking ** 100 °C/1.5 h	Sous-Vide Method 55 °C		Sous-Vide Method 65 °C		
			4 h	24 h	4 h	24 h	
Fat (%)	6.85 ^a^ ± 0.77	6.65 ^a^ ± 0.19	3.60 ^b^ ± 0.33	4.40 ^b^ ± 0.07	4.95 ^b^ ± 0.58	4.93 ^b^ ± 0.55	0.003
Water (%)	70.60 ^a^ ± 0.28	70.85 ^a^ ± 0.14	73.85 ^b^ ± 0.16	73.76 ^b^ ± 0.35	73.50 ^b^ ± 0.41	73.10 ^b^ ± 0.20	0.030
Protein (%)	20.86 ± 0.98	20.95 ± 0.07	19.90 ± 0.49	19.95 ± 0.07	20.05 ± 0.98	20.20 ± 0.55	0.250

^a,b^—Values marked with different letters differ significantly in relation to the type of thermal processing—*p* < 0.05; *—cooking until the internal temperature at the geometric center reached 85 °C, **—cooking in vacuum-sealed foil bag in water at 100 °C for 1.5 h.

**Table 2:** Physicochemical characteristics of foal meat based on the type of thermal processing (means ± SE).

Specification	Type of Thermal Treatment						
	Cooking * 85 °C	Cooking ** 100 °C/1.5 h	Sous-Vide Method 55 °C		Sous-Vide Method 65 °C		*p*-Value
			4 h	24 h	4 h	24 h	
TBARS [mg MDA/kg]	0.88 ^b^ ± 0.01	1.04 ^a^ ± 0.01	0.87 ^b^ ± 0.01	0.89 ^b^ ± 0.01	0.88 ^b^ ± 0.01	0.92 ^b^ ± 0.01	0.030
Oxidation–reduction potential [mV]	392.20 ± 6.99	356.20 ± 7.24	360.00 ± 8.76	339.50 ± 7.55	357.20 ± 8.20	357.30 ± 6.76	0.370
Water activity	0.995 ± 0.01	0.998 ± 0.02	0.997 ± 0.02	0.990 ± 0.01	0.994 ± 0.02	0.997 ± 0.02	0.290
Weight loss [%]	41.19 ^a^ ± 1.23	39.04 ^a^ ± 1.09	12.95 ^b^ ± 0.67	18.04 ^b^ ± 0.88	20.18 ^b^ ± 0.87	32.72 ^a^ ± 1.02	0.002

^a,b^—Values designated with distinct letters exhibit significant differences across the various methods of thermal treatment—*p* < 0.05; *—cooking until the internal temperature at the geometric center reached 85 °C, **—cooking in vacuum-sealed foil bag in water at 100 °C for 1.5 h.

**Table 3:** Color parameters and the level of pigments in foal meat depending on the type of thermal treatment (means ± SE).

Specification	Type of Thermal Treatment						
	Cooking * 85 °C	Cooking ** 100 °C/1.5 h	Sous-Vide Method 55 °C		Sous-Vide Method 65 °C		*p*-Value
			4 h	24 h	4 h	24 h	
L*	47.93 ^a^ ± 1.64	46.51 ^a^ ± 1.81	52.06 ^b^ ± 1.53	51.88 ^b^ ± 0.97	54.22 ^b^ ± 1.51	53.92 ^b^ ± 1.16	0.020
a*	11.53 ^a^ ± 0.08	10.65 ^a^ ± 0.51	26.32 ^b^ ± 1.51	23.25 ^b^ ± 0.70	18.02 ^c^ ± 0.26	14.34 ^c^ ± 0.81	0.003
b*	12.70 ± 0.04	13.23 ± 1.29	13.69 ± 0.42	12.51 ± 0.23	13.88 ± 0.29	13.20 ± 0.51	0.500
Mb [%]	49.49 ^a^ ± 0.24	48.44 ^a^ ± 1.19	35.23 ^b^ ± 1.68	38.57 ^b^ ± 1.17	42.25 ^a^ ± 0.09	47.83 ^a^ ± 0.28	0.010
MMb [%]	29.84 ± 0.99	29.67 ± 3.69	38.23 ± 2.02	37.64 ± 4.16	28.57 ± 0.22	27.09 ± 0.94	0.350
Mb•O_2_ [%]	20.65 ± 0.74	21.88 ± 4.39	26.52 ± 1.44	26.48 ± 3.00	21.17 ± 0.15	22.06 ± 0.66	0.270
OZB [mg/g]	585.36 ^a^ ± 2.06	528.36 ^a^ ± 10.22	305.25 ^b^ ± 4.92	410 ^c^ ± 1.25	310.69 ^b^ ± 0.50	416.38 ^c^ ± 4.85	0.0001

^a,b,c^—Values indicated by different letters show statistically significant differences across the types of thermal treatments—*p* < 0.05; *—cooking until the internal temperature at the geometric center reached 85 °C, **—cooking in vacuum-sealed foil bag in water at 100 °C for 1.5 h. Mb—myoglobin; MMb—metmyoglobin; Mb•O_2_—oxymyoglobin; OZB—total heme pigment content.

**Table 4:** Texture parameters of foal meat based on the type of thermal processing (means ± SE).

Specification	Type of Thermal Treatment						
	Cooking * 85 °C	Cooking ** 100 °C/1.5 h	Sous-Vide Method 55 °C		Sous-Vide Method 65 °C		*p*-Value
			4 h	24 h	4 h	24 h	
Shear force [N/cm^2^]	77.82 ^a^ ± 5.99	74.55 ^a^ ± 7.40	61.05 ^b^ ± 6.26	52.86 ^c^ ± 5.92	58.20 ^b^ ± 6.26	48.39 ^c^ ± 7.42	0.001
Hardness 1 [N]	113.37 ^a^ ± 14.55	112.0 ^a^ ± 13.10	106.58 ^b^ ± 9.57	102.44 ^c^ ± 12.10	105.59 ^b^ ± 9.02	101.27 ^c^ ± 9.39	0.015
Hardness 2 [N]	94.14 ^a^ ± 7.99	93.94 ^a^ ± 8.29	89.10 ^b^ ± 8.08	83.77 ^c^ ± 9.93	87.49 ^b^ ± 7.90	80.87 ^c^ ± 9.44	0.023
Stiffness 5 [N]	21.55 ^a^ ± 0.35	26.86 ^a^ ± 0.51	18.26 ^b^ ± 0.37	9.94 ^c^ ± 0.38	15.42 ^b^ ± 0.48	8.19 ^c^ ± 0.37	0.0001
Stiffness 8 [N]	57.88 ^a^ ± 2.82	56.37 ^a^ ± 1.92	53.99 ^b^ ± 2.31	45.53 ^c^ ± 2.88	51.41 ^b^ ± 2.14	42.89 ^c^ ± 2.31	0.034
Adhesiveness [mJ]	0.13 ± 0.01	0.10 ± 0.01	0.25 ± 0.02	0.22 ± 0.02	0.21 ± 0.03	0.20 ± 0.01	0.700
Cohesiveness	0.47 ± 0.05	0.52 ± 0.02	0.42 ± 0.01	0.41 ± 0.05	0.38 ± 0.03	0.41 ± 0.01	0.590
Springiness [mm]	5.12 ± 0.15	5.08 ± 0.14	4.82 ± 0.15	4.60 ± 0.10	4.50 ± 0.15	4.43 ± 0.19	0.280
Resilience	0.18 ± 0.03	0.15 ± 0.01	0.14 ± 0.02	0.13 ± 0.03	0.19 ± 0.03	0.13 ± 0.03	0.460
Chewiness [mJ]	295.23 ^a^ ± 8.65	267.13 ^a^ ± 8.70	183.0 ^b^ ± 8.09	152.43 ^b^ ± 5.56	185.80 ^b^ ± 9.01	176.30 ^b^ ± 7.69	0.0002

^a,b,c^—Values designated by different letters indicate statistically significant differences among the types of thermal processing—*p* < 0.05; *—cooking until the internal temperature at the geometric center reached 85 °C, **—cooking in vacuum-sealed foil bag in water at 100 °C for 1.5 h.

## Data Availability

The original contributions presented in the study are included in the article, further inquiries can be directed to the corresponding authors.
